# From Lab to Farm: Elucidating the Beneficial Roles of Photosynthetic Bacteria in Sustainable Agriculture

**DOI:** 10.3390/microorganisms9122453

**Published:** 2021-11-28

**Authors:** Sook-Kuan Lee, Huu-Sheng Lur, Chi-Te Liu

**Affiliations:** 1 Institute of Biotechnology, National Taiwan University, R412, No.81, Chang-Xing St, Taipei 106, Taiwan; D06642002@ntu.edu.tw; 2 Department of Agronomy, National Taiwan University, No.1, Sec. 4, Roosevelt Road, Taipei 106, Taiwan; lurhs@ntu.edu.tw; 3 Agricultural Biotechnology Research Center, Academia Sinica, No.128, Sec. 2, Academia Road, Nankang, Taipei 115, Taiwan; 4 Department of Agricultural Chemistry, National Taiwan University, No.1, Sec. 4, Roosevelt Road, Taipei 106, Taiwan

**Keywords:** phototrophic bacteria, plant growth promoting rhizobacteria (PGPR), biotic and abiotic stress, sustainable agriculture, secondary metabolite, biofertilizer, biostimulant, food crops

## Abstract

Photosynthetic bacteria (PSB) possess versatile metabolic abilities and are widely applied in environmental bioremediation, bioenergy production and agriculture. In this review, we summarize examples of purple non-sulfur bacteria (PNSB) through biofertilization, biostimulation and biocontrol mechanisms to promote plant growth. They include improvement of nutrient acquisition, production of phytohormones, induction of immune system responses, interaction with resident microbial community. It has also been reported that PNSB can produce an endogenous 5-aminolevulinic acid (5-ALA) to alleviate abiotic stress in plants. Under biotic stress, these bacteria can trigger induced systemic resistance (ISR) of plants against pathogens. The nutrient elements in soil are significantly increased by PNSB inoculation, thus improving fertility. We share experiences of researching and developing an elite PNSB inoculant (*Rhodopseudomonas palustris* PS3), including strategies for screening and verifying beneficial bacteria as well as the establishment of optimal fermentation and formulation processes for commercialization. The effectiveness of PS3 inoculants for various crops under field conditions, including conventional and organic farming, is presented. We also discuss the underlying plant growth-promoting mechanisms of this bacterium from both microbial and plant viewpoints. This review improves our understanding of the application of PNSB in sustainable crop production and could inspire the development of diverse inoculants to overcome the changes in agricultural environments created by climate change.

## 1. Introduction

In the 20th century, the emergence of the green revolution triggered a worldwide boom in the agriculture industry [1]. To feed the global population, this revolution contributed significantly to improving the productivity of crops by increasing the use of chemical fertilizers, pesticides and other agrochemicals [2]. However, the amount of arable land has drastically decreased since the start of the green revolution [3]. The overuse of synthetic agrochemicals to enhance crop yields acidifies soils, decreases fertility, destroys food web systems, pollutes air and water and releases greenhouse gases, thereby posing hazards to human health and environments [1,4,5,6,7,8]. By 2050, the world population is estimated to reach 9.8 billion, which has been estimated as Earth’s maximum capacity [9]. The combination of all these problems and challenges poses a serious threat to global food security and the stability of economies [10].

The pursuit of quality, fresh, nontoxic and safe products has become the trend of global agricultural production. To mitigate the overuse of synthetic agrochemicals and soil biodiversity loss, various beneficial microbes have been used to date as biofertilizers and biopesticides for sustainable agricultural farming [11,12]. Kloepper and colleagues first proposed the concept of plant growth-promoting rhizobacteria (PGPR), indicating that beneficial soil bacteria colonize the rhizosphere [13]. Currently, dozens of species and many hundreds of potential microbial strains of PGPR have been screened and evaluated under laboratory, greenhouse and field conditions [14]. These PGPR include diverse genera of bacteria, such as *Rhizobium*, *Pseudomonas*, *Azospirillum*, *Azotobacter* and *Bacillus* [15,16]. PGPR inoculants can fertilize several important agronomic plants, such as rice, maize, potato, bean, strawberry, cucumber and tomato [17,18,19,20,21,22]. PGPR stimulate plant growth through nutrient acquisition, biological nitrogen fixation (BNF), phytohormone production, disease control through antagonism, induced systemic resistance (ISR) or volatile organic compounds (VOCs) [13,23,24,25,26].

Photosynthetic bacteria (PSB), including oxygenic and anoxygenic phototrophic bacteria, are prokaryotes that are capable of carrying out photosynthesis [27]. Oxygenic photosynthesis is limited to cyanobacteria, whereas anoxygenic phototrophs are widely distributed among bacterial taxa. In this review, we focused on the application of anoxygenic PSB in agriculture. These bacteria can grow in either aerobic or anaerobic conditions and can use either organic or inorganic substances as electron donors to fix N_2_ and CO_2_ [28,29,30]. Among anoxygenic PSB, purple non-sulfur bacteria (PNSB) are a major group, containing *Rhodopseudomonas* spp., *Rhodobacter* spp. and *Rubrivivax* spp. [31]. They are widely distributed in natural environments, such as lakes, lagoons, wastewater ponds, sediment, moist soils, wetland ecosystems, marine environments and hypersaline systems [32,33,34]. Since PNSB possess versatile metabolic pathways, they are attractive candidates for multiple industrial applications. For example, they are broadly applied in the fisheries industry [35] and livestock industry, in bioremediation for sewage and heavy metals [36,37] and in the production of biofuels (photohydrogen or electricity) [38,39].

In addition to environmental applications, many studies have reported that PNSB can be applied directly to soil or plants to improve soil fertility and crop yield. In the first part of this review, we introduce the concept and roles of beneficial PNSB inoculants in agriculture. In the second part, the deduced mechanisms of plant growth promotion by PNSB are discussed, including the influence of PNSB on plant health and soil microbial community. In the third part, we emphasize the influence of *Rhodopseudomonas* spp. on crop production and share experiences gained while researching and designing an elite phototrophic bacterium, *Rhodopseudomonas palustris* PS3, in our lab. Overall, the information generated from this review could be very beneficial to those who are concerned about environmental protection and agricultural sustainability.

## 2. Plant Growth Promoting (PGP) Traits Exerted by PNSB on Crops

Previously, Sakarika and colleagues made a comprehensive survey in respect to the potential of using PNSB in plant production [40]. We further updated the latest studies published within these two years and summarized them in Supplementary Table S1. As shown, PNSB microbial inoculants could not only increase the yield and quality of edible plant biomass, but also alleviate the biotic and abiotic stress in crops and mitigate environmental stress. It has been demonstrated that the addition of PNSB by foliar spray or soil application significantly improves the growth responses of leafy crops, such as pak choi (*B. chinensis* L.), mustard spinach, sugar leaf, spinach and lettuce [41,42,43,44,45,46,47,48]. Similarly, the use of *Rhodobacter sphaeroides* NR3 enhanced the carotenoid content of spinach (14–138%) and mustard spinach (4.1–21%) [44]. The nitrogen content of rice grains increased by 7.1% after inoculation with *Rhodobacter capsulatus* DSM 155 [49,50]. In comparison with uninoculated treatment, *R. sphaeroides* KE149 inoculation markedly improved cucumber morphological characteristics by up to two times through IAA and organic acid production [51]. Finally, inoculation with *R. palustris* PS3 reduced the nitrate content of the nitrate-rich vegetables pak choi (20–50%) and lettuce (27%) [41]. This could have a positive effect on high-nitrate diets (e.g., Mediterranean or Japanese diets), since high dietary nitrate intake is often associated with health risks [52].

In addition to increasing the quantity and quality of leafy vegetables, inoculation with PNSB also exerts positive effects on fruits. For example, Lee (2009) reported that inoculation with *Rhodopseudomonas* sp. BL6 and KL9 resulted in the stimulation of metabolic activity and an increase in fruit weight, fruit formation and lycopene contents of tomato plants [53,54]. Kondo’s research group reported that the application of *R. sphaeroides* enhanced the quality of tomato plants, e.g., by increasing the Brix sugar content and ascorbic acid, carotenoid and citric acid contents, although the yield was not increased accordingly [55]. It has also been reported that inoculation of PSB on citrus fruit resulted in an improvement of the taste as well as an increase in the sugar and carotenoid contents (by 5.8% and 20%, respectively) [56]. Li’s research group applied a foliar spray of PNSB on melon seedlings and found that the levels of some biochemical substances, such as sucrose, soluble sugar and proline, were increased by up to 40% [57]. In addition, the biomass of PNSB-treated melon seedlings was significantly increased even under low temperatures [57]. Foliar spraying of PNSB under low temperatures also improved the chlorophyll content, photosynthetic rate and protective enzyme activity in yellow-skinned watermelon [58]. Stevia, with the common name sweet leaf or sugar leaf, is a plant that serves as a natural sweetener. Treating stevia with *Rhodopseudomonas* sp. ISP-1 by foliar spray or soil irrigation resulted in a 77–116% increase in soluble sugars and a 69% increase in stevioside content [46,47]. Moreover, it has also been reported that foliar inoculation with ISP-1 may have improved net photosynthesis and plant biomass in pot experiments.

Asian rice (*Oryza sativa* L.), which is a staple for over half of the global population, is cultivated universally [59]. In an early study, treating PNSB inoculants at the reproductive stage with N fertilizer in the form of ammonia chloride was shown to increase the grain yield of rice by 32% [60]. *R. capsulatus* DSM155 was initially isolated from a sewage processing plant [61]. When rice was inoculated with this bacterium through a hydroponic system, there was a 9–53% improvement in the aerial biomass compared to that of rice in a control treatment without inoculation [61]. Harada and colleagues reported that the *R. palustris* strain KN122 isolated from paddy soil was able to utilize rice straw as a nutrient for growth [62]. Triplicate treatments of soil and rice straw with this bacterium (dosage concentrations of approximately 5.1 × 10^9^ to 1.7 × 10^11^ MPN/pot) increased grain yields by up to 29% [62]. PNSB was also reported to increase panicle formation and plant nitrogen content. Yoshida and colleagues reported that treating rice seedlings with a PNSB inoculant three weeks before the heading stage increased the grain yield and panicle number by 200% compared to that in treatments without PNSB inoculation [63]. It has also been reported that inoculating rice with *R. capsulatus* could increase the nitrogen contents in straw and grain and the biological and grain yields of inoculated plants have been shown to be higher than those of plants without inoculation [64]. Sakpirom and colleagues reported that treating rice seedlings with the formulated *R. palustris* TN110, *Ru. gelatinosus* TN414 or mixed inoculants significantly increased the weight and length [65].

Inoculation of PNSB also has great potential to alleviate many of the abiotic stresses related to climate change in plants, such as salt stress and contamination of heavy metals [66,67]. For example, Ge and colleagues reported that treating cucumber seedlings with *R. palustris* strain G5 (isolated from the mud and water of the Qingshui River in Zhoukou city, Henan Province, China) could not only reduce the damage caused by cadmium and salt stress but also enhance the agronomic traits and activities of reactive oxygen species of the plants [68,69]. *R. palustris* CS2 and *R. faecalis* SS5 were both isolated from fish ponds in Pakistan [70]. It has been reported that these strains can detoxify arsenic (As) contamination and support seedling growth of *Vigna mungo*. *R. palustris* C1 was isolated from an As-contaminated paddy field near mines in Thailand [71]. This bacterium was reported to ameliorate As toxicity and reduce As uptake in rice. On the other hand, mixing *R. palustris* C1 and another PNSB strain, *Ru. benzoatilyticus* C31, was shown to potentially enhance the growth of two rice cultivars under As stress [72]. It has been suggested that the beneficial effects of these strains were due to the increase in the production of chlorophyll *a* and *b* resulting from the mixed culture as well as the increase in the activities of nonenzymatic (carotenoids, lipid oxidation-related and nitric oxide) and antioxidant (superoxide dismutase, ascorbate peroxidase, catalase and glutathione reductase) enzymes in rice [70,72].

Studies have indicated that PNSB play a role in plant disease control by mitigating biotic stresses. Su and colleagues reported that preinoculation of *R. palustris* GJ-22 (isolated from activated sludge) on tobacco could elevate plant immunity during subsequent tobacco mosaic virus (TMV) infection and reduce the infection rate of TMV on leaves [73,74,75]. *R. palustris* KTSSR54 (isolated from Kan-Tulee peat swamp forest, Thachana district, Surat-Thani Province, Thailand) was shown to protect rice from infection by fungal pathogens, particularly those cultivated in acidic soils [76,77]. Recently, a IAA-producing rhizobacterium *R. sphaeroides* KE149 was reported to be able to improve adzuki bean’s morphological characteristics as well as to regulate phytohormone content under flood and drought stress [78]. In addition, co-inoculation with KE149 and biochar could help to promote plant growth and strengthen the antioxidant system of soybean plant while grown under normal and stress conditions [79].

## 3. Deduced PGP Mechanisms of PNSB

**Figure 1:**
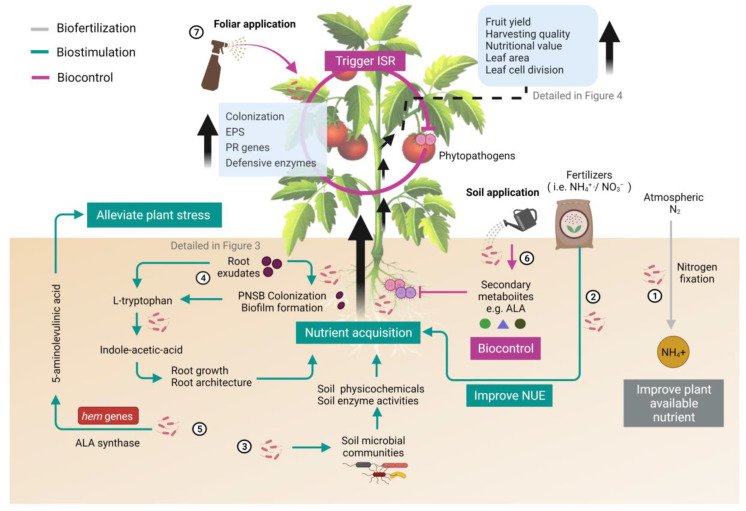
Major mechanisms by which inoculation with purple non-sulfur bacteria (PNSB) benefit the growth and quality of plants. Biofertilization (grey), biostimulation (green) and biocontrol (magenta) are three modes of action exerted by PNSB to promote plant growth and quality. The numbers marked in the illustration represent the beneficial effects exerted by PNSB, which were explained below. (1) Plant-available nutrients in soil are enhanced through biological nitrogen fixation (BNF) by free-living PNSB (biofertilization). (2) PNSB improve the nitrogen use efficiency (NUE) of host plants and stimulate plants to uptake more nitrate from soil (biostimulation). (3) PNSB interact with resident microbial community to improve soil health, thus, increase nutrient acquisition (biostimulation). (4) Indole-acetic acid (IAA) is synthesized by PNSB to promote growth and improve nutrient acquisition/absorption in roots (biostimulation). (5) 5-Aminolevulinic acid (ALA) produced by PNSB through the involvement of ALA synthase and *hem* genes to alleviate abiotic stress (biostimulation). (6) Secondary metabolites (e.g., 5-ALA) are synthesized to suppress soil pathogens directly (biocontrol). (7) Exopolysaccharides (EPSs) are produced by PNSB and pathogenesis-related (PR) genes of plants are upregulated to trigger an induced systemic response (ISR) to control plant pathogens (biocontrol). This illustration was created at BioRender.com (accessed on 20 November 2021) and the tomato image was attributed by blueringmedia (accessed on 20 November 2021).

It has been reported that PGPR improve plant growth by facilitating nutrient acquisition and phytohormone production or inducing the immune system in plants [80]. Sakarika and colleagues have summarized three key performance indicators (KPIs) of PNSB for plant production, including direct and indirect fertilization as well as biostimulation and biofortification [40]. In this review, we further proposed the PGP mechanisms of PNSB from both microbial and plant viewpoints based on the latest research findings. As illustrated in Figure 1, PNSB mainly use biofertilization, biostimulation or biocontrol to promote plant growth.

### 3.1. PNSB as Biofertilizers to Increase Plant-Available Nutrients in Soil

Nitrogen is the most abundant element in our planet’s atmosphere and is a crucially important component for all life [81]. Two of the major components of plants are chlorophyll, the most important pigment for photosynthesis and amino acids, the key building blocks of proteins [82,83]. However, atmospheric nitrogen (N_2_) in gaseous form cannot be utilized by living organisms. It has to be transformed into an available form through a process called fixation. The process of converting atmospheric nitrogen to plant-available nitrogen, that is, biological nitrogen fixation (BNF), is carried out by nitrogen-fixing bacteria [84,85]. These nitrogen-fixing bacteria are either free-living or symbiotic diazotrophs [86]. PNSB belong to free-living diazotrophs. In general, free-living nitrogen-fixing bacteria do not penetrate root tissues; instead, fixed nitrogen is taken up by associated plants, allowing better nitrogen absorption [87]. PNSB transform atmospheric molecular nitrogen into ammonia (NH_3_) or ammonium (NH_4_^+^) with a specific nitrogenase enzyme, thus making nitrogen available for plant absorption [84,88]. Since biofertilizers are living microbes that can enhance plant nutrition by mobilizing or increasing nutrient availability, PNSB can be categorized among them. Previously, nitrogen fixing ability was determined by Nessler’s reagent and a nitrogen-free medium without nitrogenase cofactors [65]. Sakpirom and colleagues reported that the elite *R. palustris* strain TN110 encoded three sets of Mo, V and Fe nitrogenase gene clusters, which resulted in the release of higher concentrations of NH_4_^+^ than other tested strains [65]. On the other hand, although the elite *R. palustris* strain PS3 could fix N under light-microaerobic condition, only *anf* (encoding iron nitrogenase) and *nif* (encoding molybdenum nitrogenase) nitrogenase-related genes and no vnf-related gene were found in its genome [89].

### 3.2. PNSB as Plant Biostimulants or Growth Regulators

Plant biostimulants (PBs) are a new category of crop inputs, which have been attracted broad attention for the past decade. They are recently under the new Regulation (EU) 2019/1009, which led to the following: “A plant biostimulant shall be an EU fertilizing product the function of which is to stimulate plant nutrition processes independently of the product’s nutrient content with the sole aim of improving one or more of the following characteristics of the plant or the plant rhizosphere: (i) nutrient use efficiency, (ii) tolerance to abiotic stress, (iii) quality traits, or (iv) availability of confined nutrients in the soil or rhizosphere” (EU, 2019). Some PNSB inoculants functions in accordance with these criteria are described below.

#### 3.2.1. Indole-3-Acetic Acid (IAA) Production by PNSB

Phytohormones are key participants in regulating plant growth and development [90,91]. Many beneficial soil bacteria are known to secrete phytohormones for root uptake or to regulate hormone balances in crops, thus enhancing growth and abiotic responses [92].

Because they control assorted stages of plant growth and development, auxins play key roles in regulating processes of the plant life cycle, such as cell division, cell elongation, tissue differentiation and apical dominance [87,93]. Indole-3-acetic acid (IAA) is the most widely studied auxin produced by PGPR [1] The IAA produced by PGPR stimulates seed germination and root development; enhances vegetative growth and fructification; improves photosynthetic and biosynthetic abilities, such as the production of pigments and metabolites; elicits transcriptional differences in hormone-, defense- and cell wall-related genes; and is responsible for alleviating the abiotic stress of plants, such as that due to drought and saline [91,93,94,95,96]. Biosynthesis of IAA in microorganisms occurs via tryptophan-dependent and the tryptophan-independent pathways [97,98]. In general, PNSB can produce IAA through indole-3-pyruvate (IPA) and tryptamine (TAM) pathways, which use tryptophan as a precursor molecule [54,99]. However, there are few studies on the tryptophan-independent pathway in bacteria [98]. Mariana and colleagues suggested that the tryptophan-independent pathway is made up of three stages [100]: from chorismic acid (CHA) to anthranilic acid (AA), from AA to IAA and from tryptophan (TRP) to AA through kynurenine (KYN). For the elite *R. palustris* PS3 strain described above, IAA (140 µM/OD_530_) can be produced in the presence of tryptophan and synthesized via an unidentified pathway with some genes, such as *trpBA* and *tnaA* genes, which were involved in the IAA synthesis [89]. Sakpirom and colleagues found that *R. palustris* TN110 and *Rubrivivax gelatinosus* TN41 could produce 0.65–3.6 mg/L IAA under facultative aerobic conditions [65]. Two other *R. palustris* strains, GJ-22 and KL9, have also been reported to yield 30–52 mg/L IAA in the presence of tryptophan [73,101]. It was reported that *R. palustris* KKSSR91, which secreted IAA (29.58 mg/L) in the presence of 3 mM tryptophan, was able to reduce the phytotoxic effects of acid on kidney bean plants [102]. *Rhodobacter capsulatus* PS-2, which was isolated from paddy soil, was capable of producing a large amount of IAA (197.44 ± 5.92 mg/L) in an optimal medium containing 0.3% tryptophan. [103]. Recently, Kang and colleagues also reported that *R. sphaeroides* KE149 could produce 4.6–5.3 μg/mL of IAA in the presence of D-tryptophan in medium and was able to alleviate the adverse effect of water stress for adzuki bean plants [78].

#### 3.2.2. PNSB Improve Nitrogen Use Efficiency by Interaction with Plants

Nitrogen use efficiency (NUE) reflects the potential ability of plants to consume and utilize nitrogen for maximum yields [104]. NUE is associated with both N uptake efficiency (NupE) and N utilization or assimilation efficiency [104,105,106]. Increasing NUE is critical to improve crop yield, reduce N fertilizer demand and alleviate environmental pollution. PNSB has been reported to improve the NUE of plants [41,42,45]. Elbadry and Elbanna found that inoculating rice with *R. capsulatus* DSM155 in the presence of N fertilizer could increase the nitrogen content in roots by 20%. However, the effect of inoculation was lower in the presence of N fertilizer than in N-deficient conditions [61]. In addition, inoculation with DSM155 caused a 2.5-fold increase in N contents in roots under N-deficient conditions [61]. Thus, these results suggested that *R. capsulatus* DSM155 could not only supply the host plants with fixed nitrogen in the absence of N fertilizer but also improve the NUE in the presence of N fertilizer [61].

Excessive application of nitrogen fertilizer is considered the major cause of nitrate accumulation in plants [107]. In particular, leafy vegetables tend to accumulate nitrate under low light conditions, as the uptake of nitrate exceeds the rate of nitrate reduction [108]. High nitrate contents in vegetables potentially increase the risk of human illnesses, such as gastric cancer, esophageal cancer and methemoglobinemia [109]. Wong and colleagues reported that the nitrate contents in the leaves of Chinese cabbage were significantly lower in plants in which the roots were inoculated with *R. palustris* PS3 than in plants that were not inoculated [45]. Furthermore, Hsu and colleagues demonstrated that the NUE of plants was remarkably increased in the presence of PS3 inoculation, allowing nitrate to be effectively catabolized [42].

#### 3.2.3. 5-ALA of PNSB Can Alleviate Abiotic Stress of Plants

Drought, salinity and extreme temperature were thought to contribute to 70% of yield gap dictated by global climatic change [110]. Given the current climate change scenario, abiotic stresses are likely to pose a serious threat to crop productivity and, thus, global food security [111]. In order to address this situation, the application of plant biostimulants has been proposed as one of the most promising and efficient strategies for improving yield stability [112].

5-Aminolevulinic acid (5-ALA), which is abundant in bacteria, algae, plants and animals, is an intermediate compound involved in tetrapyrrole biosynthesis of compounds such as porphyrin, heme, chlorophyll and vitamin B12 [113]. 5-ALA is also considered a plant growth regulator, participates in the enhancement of plant growth and yield and confers tolerance to plants of various abiotic stresses [46]. Exogenous 5-ALA has been reported to increase crop yields by regulating the chlorophyll biosynthesis and photosynthesis systems [114,115]. However, the underlying mechanisms through which 5-ALA regulation of plant growth have not been fully elucidated. The use of PNSB has been reported to be an effective approach for ALA production [116]. Many PNSBs, such as *R.* palustris, *R. sphaeroides* and *Rhodovulum* sp., have been identified as potential 5-ALA producers [65,117,118,119]. According to the results of comparative genomics, the genes *hemO* and *hemA* of *R. palustris* are associated with the biosynthesis of 5-ALA [89]. Nunkaew and colleagues reported that the application of *R. palustris* TN114 supernatant ameliorated rice seedling growth under NaCl stress and the effect was comparable to that of commercial 5-ALA [118]. 5-ALA can also be used as a safe and biodegradable herbicide [120,121].

#### 3.2.4. PNSB Interaction with Microbial Communities to Improve Soil Health and Crop Quality

Soil health refers to the ecological equilibrium and functionality of soil, indicating the ability to sustain agricultural productivity and protect environmental resources [122]. In general, healthy soils contain favorable biological, physical and chemical properties for producing healthy crops [94,123]. The indicators for evaluating soil health include those of soil microbial communities and physiochemical and enzyme activities [122,124,125]. Beneficial rhizosphere microbes play vital roles in organic matter decomposition and nutrient cycling of carbon, nitrogen and phosphorus; these processes may represent a correlation between plant and soil functions [126,127]. Generally, soil enzyme activities directly reflect the metabolic requirements and available nutrients of soil microorganisms [128]. As mentioned, the structures of soil microbial communities can be influenced by the physical and chemical properties of the soil as well as by exotic microbial inoculants [129,130,131]. Microbial inoculants must sustain their populations and interact with indigenous microbes in soil to enhance plant growth [132]. Xu and colleagues found that inoculating stevia (i.e., sugar leaf) in continuously fertilized soil with *R. palustris* elevated the soil dehydrogenase and urease activities to a remarkable extent [47]. Moreover, the abundances of some bacterial lineages in the soil increased. It has been indicated that continuous use of chemical fertilizers has strong negative effects on soil microbial community properties, especially on dehydrogenase and urease activities [133]. Therefore, *R. palustris* inoculation can improve soil health by reducing the negative impacts of continuous chemical fertilization on the microbial community [47]. This research group also reported that treating Chinese pak choi with *R. palustris* can enhance soil microbial metabolic activity and alter the abundances of some bacterial groups [47,48]. They found that the abundances of the operational taxonomic units (OTUs) related to the acceleration of carbon and nutrient cycling in soil, such as those phyla belonging to the Proteobacteria, Planctomycetes, Acidobacteria, Actinobacteria, Verrucomicrobia and Nitrospirae, were significantly increased following treatment with *R. palustris*. Lee’s group reported that the application of PNSB improved the fresh weights and lycopene contents of tomatoes; however, the bacterial communities in the rhizosphere were not altered [53]. Wang and colleagues reported that inoculating peanut with a mixed bacterial inoculant containing *R. palustris* ISP-1 and *Burkholderia rabicn* ISOP5 improved yield and increased soil fertility and metabolic activity [134]. These authors conducted gene functional analysis with PICRUSt 1.1.4 and found that the abundances of the genes associated with inorganic P solubilization, organic P mineralization and N metabolism was remarkably increased. They noticed that the abundance of the phylum Verrucomicrobia was significantly increased in this treatment [134]. It has been deduced that the bacteria belonging to Verrucomicrobia are associated with soil fertility, which plays an important role in the degradation of organic matter [134,135]. Taken together, these results shown that inoculation with PNSB could improve soil fertility and enzymatic activities by altering the abundances of related bacteria and functional gene expression.

### 3.3. PNSB as Biological Control Agents

Biocontrol refers to the use of other organisms to control pests, like insects, mites, weeds and plant diseases [136]. Plant diseases cause damage to crop yield, reproduction, photosynthetic activity and growth [137]. In addition to using chemical pesticides, plant diseases can be controlled through biocontrol, which is regarded as an environmentally friendly alternative approach to suppress plant pathogens through the application of living organisms [138]. PGPR also play a major role in biocontrol by producing pathogen-antagonistic substances and/or by inducing systematic resistance in plants to pathogens [139]. PGPR-elicited ISR enhances the defensive capacity of host plants and reduces damage from pathogens [140]. Su and colleagues reported that inoculating *R. palustris* GJ-22 onto tobacco (*Nicotiana benthamiana*) leaves could induce ISR to TMV [73,74]. The abundance of TMV was reduced to a remarkable extent, the activities of defensive enzymes were enhanced and the transcripts of pathogenesis-related (PR) genes were upregulated [73,74]. Furthermore, such ISR has been demonstrated to be triggered by an exopolysaccharide, G-EPS, secreted by *R. palustris* GJ-22 [141].

## 4. Developing Elite PNSB Inoculants for Sustainable Agriculture

In general, several beneficial microbial traits (i.e., in vitro PGP traits), such as BNF, phosphate solubilization, 1-aminocyclopropane-1-carboxylic acid (ACC) deaminase activity and siderophore and phytohormone production, are assessed via laboratory screening assays to select elite PGPR for the development of microbial inoculants [142]. However, many reports have already indicated that the existence of these PGP microbial traits in vitro is not absolutely associated with plant growth promotion [143,144]. Taking PNSB as an example, PS3, YSC3 and BCRC16408 (ATCC 17001^T^) are three closely related *R. palustris* strains [45]. These three strains were able to fix nitrogen under a free-living state and produce IAA in the presence of tryptophan; however, only PS3 was proven to be able to improve plant growth [45]. Furthermore, Lo and colleagues reported that the PS3 and YSC3 strains possessed very similar genome structures and genes associated with plant growth promotion; however, only PS3 showed beneficial effects on plant growth [89]. Accordingly, these findings all indicate that the in vitro presence of PGP traits or PGP-related genes does not necessarily indicate phenotypes associated with plant growth promotion.

The application of PGPR inoculants is an effective biological approach to increase crop yields. However, the effectiveness of a PGPR inoculant in the field depends on a various factors, such as environmental conditions, plant types, microbe-plant interactions and indigenous microbial communities [145]. Many studies have elucidated the mechanisms of PGPR inoculant with single strain and single host plants [146]. However, PGPR strains do not act individually in the rhizosphere but rather as part of a bacterial community [146]. PGPR populations may display antagonistic or synergistic effects, depending on their interaction with microbial communities [146]. Therefore, the complexity of interactions between PGPR and the resident microbiome needs to be considered when inoculants are applied to the field. A previous work showed that while inoculating PNSB on some plant species, Proteobacteria, Planctomycetes, Acidobacteria, Actinobacteria, Verrucomicrobia and Nitrospirae, increased in the rhizosphere [47,48]. Accordingly, they deduced that these nutrient cycle related phyla acted synergistically with the PNSB inoculant to improve plant biomass. However, there is no further physiological or molecular evidence to verify the roles of the microbial phyla in the rhizosphere. Recently, Santoyo and colleagues proposed expanding the use different microbial consortia to provide more consistent results and performance in the fields [147].

In general, the performances of PGPR inoculants under laboratory or greenhouse conditions are not easily transferred to those under field conditions, particularly when dealing with gram-negative, non-spore-forming bacteria [148]. These microbes do not form spores or go dormant and they are more susceptible than other bacteria to detrimental factors occurring during processing and field application [149,150]. Without appropriate formulations, microbial inoculants of non-spore-forming bacteria are easily damaged during processing, cannot be easily stored for long periods under harsh conditions (i.e., they have a short shelf life), or do not easily survive in the rhizosphere after application [151].

To enhance the survival rate and effectiveness of non-spore-forming bacteria in inoculants, it is necessary to develop suitable formulations [151]. PNSB are non-spore-forming bacteria and according to a previous study, most are applied to soil in liquid form. The advantages of liquid-based inoculants are their easy processing and the low costs of additive materials compared to those for solid-based formulations [152]. Common additive materials, such as coconut water, polyvinyl alcohol, xanthan gum, gelatin, mineral oil and rabic gum, can prolong cell survival during storage [153,154,155,156,157,158]. On the other hand, because liquid-based inoculants are packaged for long-term storage, they are subject to several abiotic stresses, such as nutrient depletion, extreme temperatures or hypoxia [151,159,160]. Proper formulation can mitigate those stresses during storage to a certain extent. In the case of *R. palustris*, Lee and colleagues found that horticultural oil was a safe and low-cost additive for formulations [155]. This oil can act as an additional nutrient source, allowing *R. palustris* to maintain growth during storage and after application [155]. There is also an increasingly popular formulation technique for liquid-based inoculants that involves biochar. Biochar is a charcoal-like substance that is made by burning organic material and can adsorb beneficial bacteria, as well as organic ingredients, due to its high internal porosity and large surface area [161,162,163]. It has been reported that encapsulating bacteria in or coating bacteria with biochar material could form a habitat and protect the bacteria from stressful conditions to prolong their survival [164,165,166]. Although biochar-based formulation is a potentially promising technique, in consideration of its cost as well as concern over its potential toxicity, further evaluation is needed [167].

## 5. PNSB Inoculants Can Improve the Quality and Nutritional Value of Food Crops

It has been well studied that PGPR application can also improve the quality and nutritional value of agricultural products [168]. For example, Sharma’s research group reported that the inoculation with *Pseudomonas putida*, *Pseudomonas fluorescens* and *Azospirillum lipoferum* enhanced the grain iron content in rice [169]. Kondo and colleagues reported that application of *R. sphaeroides* increased the quality of tomato plants [55]. In addition, Yildirim and colleagues demonstrated that broccoli roots inoculated with PGPR (*Bacillus cereus*, *Brevibacillus reuszeri* and *Rhizobium rubi*) could increase dietary nutritional values, such as N, K, Ca, S, P, Mg, Fe, Mn, Zn and Cu [170].

Some leafy vegetables, such as spinach, parsley, fennel and rocket, tend to accumulate high levels of nitrates and excess N supply is considered to be the major cause of nitrate accumulation [107,171,172,173]. In consideration of the undesirable effects on human health, the EU has set the regulation for the levels of nitrates in leafy vegetables [174,175]. Non-heading Chinese cabbage (*B. rapa* var. chinensis) is a popular Asian leafy vegetable with a relatively high nitrate concentration [176]. Hsu and colleagues found that while inoculating an elite PNSB strain *R. palustris* PS3 in the hydroponic nutrient solution during cultivation of non-heading Chinese cabbage, not only was the yield increased, but also the nitrate content was remarkably decreased by 88% [41]. Intriguingly, although the nitrate uptake was significantly elevated in the PS3-inoculated plants, the excess nitrate was not accumulated in the tissues [42]. This is because the plants showed high N use efficiency (NUE) in the presence of *R. palustris* PS3 [42].

PNSB has also shown the potential to enhance the contents of a variety of secondary metabolites in crops [53,79]. For example, *R. palustris* KL9 was reported to enhance the lycopene content in harvested tomato fruits. *R. sphaeroides* KE149 was reported to increase the production of flavonoids and phenolics in soybean under normal condition [79]. Both lycopene and flavonoids have been shown to have antioxidative activities, which can provide positive effects for the maintenance of health and the prevention of diseases, such as chronic diseases, including cancer, asthma, inflammation, cardiovascular disorders, etc [177,178]. Wu and colleagues reported that while treating *Stevia rebaudiana* with *Rhodopseudomonas* sp. ISP-1 resulted in a dramatic increase in the stevioside content in leaves. Stevioside is a steviol glycoside which are the secondary metabolites responsible for the sweetness of Stevia. This compound is used as a natural sweetener for type II diabetic patients and safe to consume in appropriate dosage [179,180]. These findings all indicate that PNSB can improve the quality as well as elevate the accumulation of secondary metabolites of agri-food products effectively. Taken together, we believe the application of PNSB in medicinal plants and health is worthy of further exploration.

## 6. Our Research and Development Journey to PSB as Elite PGPR Inoculants

**Figure 2:**
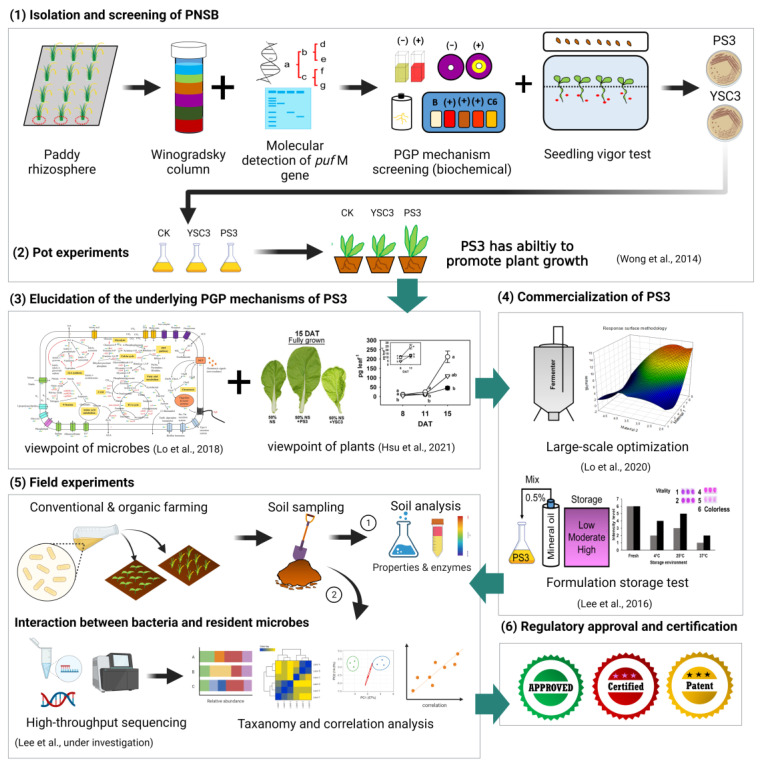
Diagram of the research and development processes of the *R. palustris* PS3 inoculant: (1) Isolate and screen of PNSB; (2) perform pot experiments to verify the PGP efficiency of isolates; (3) elucidate the underlying PGP mechanisms of PS3 from the viewpoint of microbes and plants; (4) develop optimal fermentation and formulation processes for commercialization; (5) perform field experiments to verify the efficacy and performance of the PS3 inoculant under conventional and organic farming conditions; (6) obtain regulatory approval and certification of the PS3 inoculant as a biofertilizer or plant biostimulant. This illustration was created at BioRender.com (accessed on 20 November 2021).

As described in the previous sections, PNSB have many traits that confer benefits to plant growth, such as increased crop yield and harvest quality, or tolerance to stressful environments. Since PNSB possess versatile functions, they have the potential to become elite microbial inoculants, such as biofertilizers or biostimulants. Hereinafter, we share our experiences researching and developing an *R. palustris* inoculant. The experimental strategy to develop the inoculant based on *R. palustris* PS3 is shown in Figure 2.

### 6.1. Isolation and Screening of PNSB

PNSB are widely found in nature, especially in submerged environments, such as paddy fields and sediments [181]. To efficiently isolate PNSB, we combined the conventional Winogradsky soil column and molecular marker (*pufM* gene) detection methods to enrich and isolate microbes from rice paddy fields located all around Taiwan [45]. We initially conducted in vitro screening for PGP traits to select potential isolates. However, we noticed that many isolates that exhibited relative high potency in vitro PGP traits were not necessarily able to promote plant growth (data not shown). We alternatively conducted a seedling vigor test, through which we pre-evaluate the compatibility and incompatibility of the isolates and host plants [45]. We selected several strains from the PNSB isolates and PS3 showed the highest seedling vigor index among the selected strains [45].

### 6.2. Pot Experiments

In consideration of the adverse environmental impacts of agrochemicals and to support sustainable agricultural development, many countries have set goals for reducing the application of these chemicals in agriculture [182]. In Taiwan, it has been proposed that agrochemical use should be reduced by 30–50% in the long term. Accordingly, we set a criterion to select promising PNSB inoculants that can sustain proper crop yield even under half of the conventional fertilizer dosage. We inoculated the potential PNSB isolates into soil (~10^6^ CFU/g soil) cultivated with Chinese cabbage (*Brassica rapa* L. ssp. *chinensis* var. Maruha) with half the typical rate of chemical fertilizer (50% CF). We then evaluated the agronomic characteristics of 30-day-old Chinese cabbage plants [45]. The growth of plants in the treatment with 50% CF only was markedly less than that in the treatment with the full amount of chemical fertilizer (100% CF). However, when the PS3 inoculant was added to the treatment with 50% CF, the plant biomass (fresh and dry weights of the shoots) was significantly greater than that in the treatment with 50% CF alone and statistically comparable to that in the treatment with 100% CF. On the other hand, when 50% fertilizer was combined with the other PNSB isolates, lower growth or inconsistent growth was observed compared with that in the treatment with 100% CF [45]. Many rounds of trials were conducted to confirm the growth-promoting traits of PS3 and we determined that PS3 has the potential to promote plant growth even with less fertilizer input than is typically applied. According to the results of molecular phylogenetic analysis and biochemical characterization, strain PS3 was identified as *R. palustris*, which is a facultatively phototrophic/chemotrophic PNSB [45].

### 6.3. Elucidation of the Underlying PGP Mechanisms of PS3

#### 6.3.1. From the Viewpoint of Microbes

**Figure 3:**
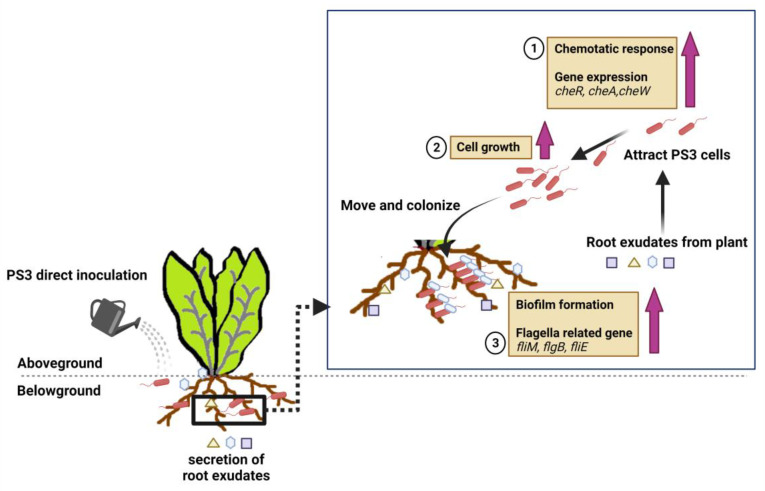
A deduced plant-growth promoting mechanism based on the viewpoint of microbes (refer to Lo et al., 2018) [89]. (1) *R. palustris* PS3 cells are attracted by the root exudate of the host plant (i.e., chemotactic response); (2) Cell growth of PS3 is induced in the presence of root exudates; and (3) PS3 cells move close to roots and colonize the surface of roots by forming biofilms. This illustration was created at BioRender.com (accessed on 20 November 2021).

Soil microorganisms play essential roles in plant growth and plant exudates by altering the internal physiological status of plants to different extents [92,183]. Root exudates may act as signaling messengers that stimulate biological and physical interactions between plant roots and soil organisms and affect the structures of microbial communities [184]. To elucidate the underlying mechanisms of the beneficial effects exerted by PS3, we explored the modes of action from the microbial and plant viewpoints. For comparative analyses, YSC3, a PGP-ineffective *R. palustris* strain, was introduced. According to a phylogenetic tree, the genetic relationship between YSC3 and PS3 was very close [45]. We conducted whole-genome sequencing analysis and found that the genomic structures of PS3 and YSC3 were very similar [89] (Supplementary Figure S1). Furthermore, both strains possessed genes associated with plant growth promotion, such as those related to nitrogen fixation, IAA synthesis and ACC deamination [89]. However, we found that when treating Chinese cabbage roots with exudate solutions containing each strain, the growth rate, amount of biofilm formation and relative expression levels of several chemotaxis-associated genes were significantly higher for the plants in the PS3 treatment than for those in the YSC3 treatment [89] (summarized in Figure 3). These results indicate that PS3 responds more sensitively than YSC3 to the presence of plant hosts, which may contribute to the successful interactions of PS3 with plant hosts. In addition, we demonstrated that the existence of gene clusters associated with PGP is required for a bacterium to exhibit phenotypes associated with beneficial effects; however, the presence of these genes does not necessarily indicate their expression [89]. Therefore, the effectiveness of PGPR is not necessarily coupled with their genetic background or in vitro characteristics.

#### 6.3.2. From the Viewpoint of Plants

The shoot biomass of PS3-inoculated Chinese cabbage was significantly higher than that of YSC3-inoculated Chinese cabbage [45], which was mainly due to the enlargement of the leaf area of young expanding leaves [42]. Since there were no significant differences in leaf number among the treatment groups, we deduced that the beneficial effect in shoot biomass was due to leaf area expansion rather than elevation in the number of leaf blades. Auxins are responsible for many aspects of plant growth and we found that the endogenous IAA level in the leaves of the PS3-inoculated plants was significantly higher than that in the YSC3-inoculated plants. Some literature has indicated that there is a positive correlation between endogenous auxin levels in host plants and exogenous auxin production by beneficial microbes [185,186]. However, exogenous auxin production by PS3 was not higher than that by YSC3 and auxin production by these strains was equal in the presence of tryptophan [45]. We further verified the bacterial expression of IAA synthesis-related genes during root colonization and found the same expression level between strains [42]. Accordingly, we deduced that auxin accumulation in the shoots of the PS3-inoculated plants was systemically induced by the root-colonizing bacterium, not due to the direct transport of bacterial auxin from roots.

In addition to biomass, we found that the N content and the NUE of PS3-inoculated Chinese cabbage were dramatically higher than those of YSC3-inoculated plants [42]. As mentioned, NUE is associated with both NUpE and N assimilation efficiency (NUtE). In the PS3-inoculated plants, the former was significantly increased; however, the latter was almost the same as in the YSC3-inoculated plants. We analyzed the transcripts of some nitrate transporter-related genes in roots and found that the expression rate of the gene encoding a low affinity nitrate transporter (NRT 1.1) was significantly higher in PS3-inoculated plants than in YSC3-inoculated plants during the expanded leaf development stage [42].

**Figure 4:**
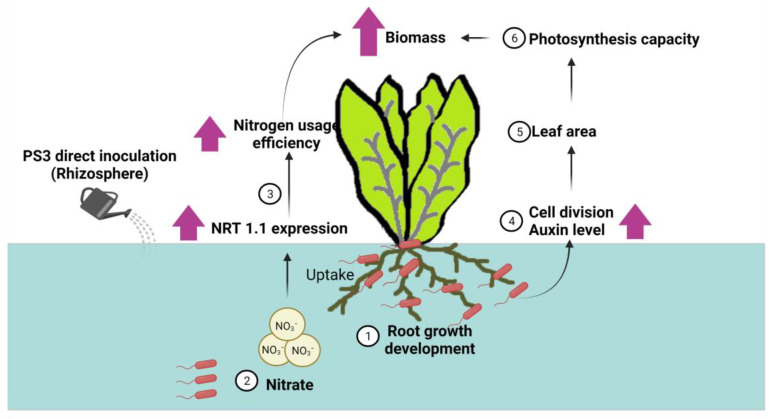
A deduced plant-growth promoting mechanism based on the viewpoint of plants (refer to Hsu et al., 2021) [42]. Here, we show the interaction between *R. palustris* PS3 and Chinese cabbage cultivated in a hydroponic system. (1) PS3 cells stimulate root growth and development; (2) nitrate uptake of Chinese cabbage is increased by PS3 inoculation (i.e., NRT1.1 transcript increased); (3) nitrogen use efficiency (NUE) of Chinese cabbage is remarkably enhanced; (4) endogenous IAA concentration as well as cell division rate are significantly increased in the young expanding leaves; (5) leaf size is remarkably enlarged; and (6) photosynthetic capacity of plant is notably increased. (Patent: method of reducing nitrite content in a plant, US10,015,935,B2). This illustration was created at BioRender.com (accessed on 20 November 2021).

Taken together, these results show that inoculation with PS3 could promote plant growth by enhancing nitrate uptake and stimulating the accumulation of endogenous auxin in young expanding leaves to increase the proliferation of leaf cells during leaf development [42] (summarized in Figure 4). We concluded that all the differences between the PS3- and YSC3-inoculated plants were due to the differences in responses elicited by compatible and incompatible bacterium-plant interactions.

### 6.4. Optimal Fermentation and Formulation

There are a number of factors, such as the large-scale of commercial manufacturing, the suitability of formulations and the methods of application, that influence the quality and efficacy of microbial inoculants during production and processing and after inoculation into soil. For large-scale commercial manufacturing, optimizing the fermentation process is an essential step. To develop an optimal fermentation protocol for *R. palustris* PS3, we evaluated the use of low-cost materials as culture media and optimized the culture conditions via the response surface methodology [187]. We developed a novel medium for *R. palustris* fermentation with agro-industrial byproducts, i.e., corn steep liquor (CSL) and molasses, as nitrogen and carbon sources [187]. In addition, we applied response surface methodology to determine the optimum fermentation process and the effectiveness of the new fermentation broth was verified by pot experiments [187]. Our newly developed medium and process for large-scale industrial production provide a prospective strategy with the benefits of cost and time effectiveness, as well as environmental sustainability.

**Figure 5:**
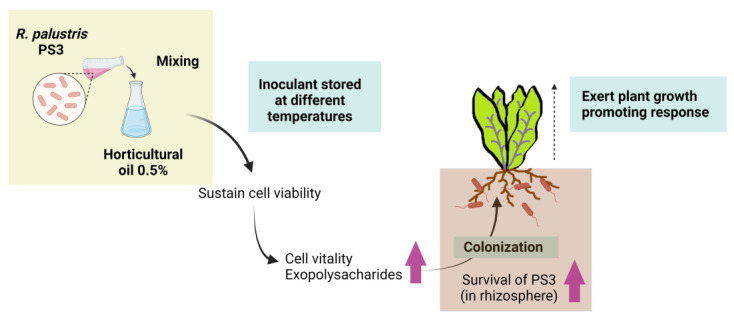
Beneficial effects of horticultural oil as a potential additive for liquid-based formulation of *R. palustris PS3* inoculant (refer to Lee et al., 2016) [155]. After supplementing 0.5% horticultural oil in the bacterial broth, both the viability (i.e., culturability) and vitality (i.e., metabolic activity) of PS3 cells were remarkably improved even after storage at relatively high temperatures. The exopolysaccharide (EPS) content in the formulated inoculants was significantly increased and was favorable for colonization on the surface of roots. When inoculated with plants, the formulated PS3 inoculants showed a greater ability to survive in soil and exert plant growth-promoting traits than the unformulated inoculants. This illustration was created at BioRender.com (accessed on 20 November 2021).

The inconsistent efficacy of PGPR inoculants in field conditions, which usually do not perform as well as those in greenhouse or laboratory experiments, has been widely discussed [188,189]. In most cases, this gap in performance is due to inadequate formulation and poor inoculant quality [190,191,192]. As mentioned, *R. palustris* are nonspore-forming bacteria that are difficult to formulate into solid-based inoculants. To develop a PS3 inoculant for practical use, we evaluated several oil- and polymer-based additives for PS3 formulation development. We analyzed the survival of PS3 in various formulations and at different storage temperatures for a set period of time and assessed the beneficial effects of these inoculants on plants grown in pots. We ultimately chose horticultural oil (0.5%) as a potential additive because it maintained a relatively large population of bacteria and conferred greater microbial vitality than other additives under various storage conditions [155]. Better plant growth-promoting effects were observed in the treatment with the formulated PS3 inoculant than in the treatment without the inoculant [155] (summarize in Figure 5). Intriguingly, we noticed that although the survival and root colonization were almost identical in the formulated and unformulated treatments, higher recovery activities during storage and greater plant growth promotion were observed in the formulated treatment than in the unformulated treatment [155]. Accordingly, to address the gaps between the expected and actual performances of PGPR inoculants, we suggest that not only the viability (i.e., cultivability) but also the vitality (i.e., metabolic activity) of bacteria should be considered indices of quality control.

### 6.5. Field Experiments

It has been reported that the efficacy and performance of PGPR inoculants under field conditions are affected by plant species and varieties, geographical and climatic conditions, soil physicochemical environments and the application of chemical fertilizers or pesticides [193,194,195]. The effectiveness of PS3 for plant growth promotion was verified in various farming systems (Supplementary Table S2). For conventional farming systems, the yield of heading Chinese cabbage inoculated with PS3 and given half the normal amount of chemical fertilizer was ~39% and ~23% higher than those that were not inoculated and given half and full amounts of chemical fertilizer, respectively. PS3 inoculation also benefited the yield of Chinese flowering cabbage (Choi Sum) and pepper leaves when these plants were cultivated under conventional farming systems with organic amendment. For organic farming systems, the performance of PS3 was determined in both cruciferous vegetables and plants belonging to Asteraceae, such as Chinese cabbage, lettuce and sesame leaves. Consumable yields of these crops significantly increased by 25–43% compared with that of the control (without inoculation). Notably, PS3 inoculation also had beneficial effects on tomato fruit growth, taste and harvesting quality in organic farming systems (Lee et al., unpublished data). The long-term changes in plant growth, the physicochemical properties of soil, soil enzymatic activities and rhizosphere microbial community dynamics caused by the application of PS3 are under investigation. We expect this study to enhance our mechanistic understanding of the interactions between plants and microbes and how these processes can be optimized to drive plant nutrition with organic fertilizers.

## 7. Conclusions and Perspective

PNSB are versatile microorganisms that are able to exert plant growth-promoting effects on crops and are discussed in this review (Section 2). In this review, we summarize examples of PNSB through biofertilization, biostimulation and biocontrol mechanisms to promote plant growth. They can improve plant growth by fixing nitrogen, facilitating nutrient acquisition and producing phytohormones. Furthermore, they are able to synthesize 5-ALA to confer abiotic stress tolerance to host plants (Section 3). Under biotic stress, they may trigger ISR and enhance bacterial colonization by beneficial bacteria to protect plants from pathogen attack (Section 3). In addition, PNSB can regulate microbial communities to enhance soil fertility elements, which consequently have beneficial effects on the nutrient use efficiency and quality of plants (Section 3). Since PNSB are nonspore-forming bacteria, their performance in the field is easily affected by environmental conditions. To enhance their survival rate and effectiveness, a suitable formulation is needed for the commercialization of these inoculants (Section 4). In addition, PNSB are found to improve the quality and trigger the accumulation of secondary metabolites of food crops (Section 5). Accordingly, it is worth further exploring the application of PNSB in medicinal plants and human health. We also share our experiences researching and developing the elite *R. palustris* PS3 inoculant (Section 6). Based on the experimental results, we suggest that not only the viability (i.e., cultivability) but also the vitality (i.e., metabolic activity) of PGPR should be considered indices of quality control for microbial inoculants. Given their beneficial effects and wide applicability to crops, PNSB are promising inoculants in sustainable agriculture and provide a solution to crop production under climate change.

## Data Availability

Not applicable.

## Supplementary Materials

The following are available online at https://www.mdpi.com/article/10.3390/microorganisms9122453/s1, Figure S1: Genes potentially related to plant growth promotion and biofilm formation in different strains of PS3, YSC3, and CGA009, Table S1: Beneficial effects of PNSB on different crops and Table S2: Plant growth responses to PS3 inoculation under different experimental conditions and farming systems.
