# Supplementary material for: From Lab to Farm: Elucidating the Beneficial Roles of Photosynthetic Bacteria in Sustainable Agriculture
Source: Microorganisms. 2021 Nov 28;9(12):2453. doi: 10.3390/microorganisms9122453 (PMC8707939; doi:10.3390/microorganisms9122453)
Supplement: Supplementary file 1 [file microorganisms-09-02453-s001.zip › microorganisms-1452842-supplementary.pdf]

microorganisms

Review

From Lab to Farm: Elucidating the Beneficial Roles of Photosynthetic Bacteria in Sustainable Agriculture

Sook-Kuan Lee [1], Huu-Sheng Lur [2] and Chi-Te Liu [1,3,4,*]

1 Institute of Biotechnology, National Taiwan University, R412, No. 81, Chang-Xing St, Taipei 106, Taiwan; D06642002@ntu.edu.tw
2 Department of Agronomy, National Taiwan University, No.1, Sec. 4, Roosevelt Road, Taipei 106, Taiwan; lurhs@ntu.edu.tw
3 Agricultural Biotechnology Research Center, Academia Sinica, No.128, Sec.2, Academia Rd., Nankang, Taipei 115, Taiwan
4 Department of Agricultural Chemistry, National Taiwan University, No.1, Sec. 4, Roosevelt Road, Taipei 106, Taiwan
* Correspondence: chiteliu@ntu.edu.tw; Tel.: +886-2-3366-6025

Supplementary Materials

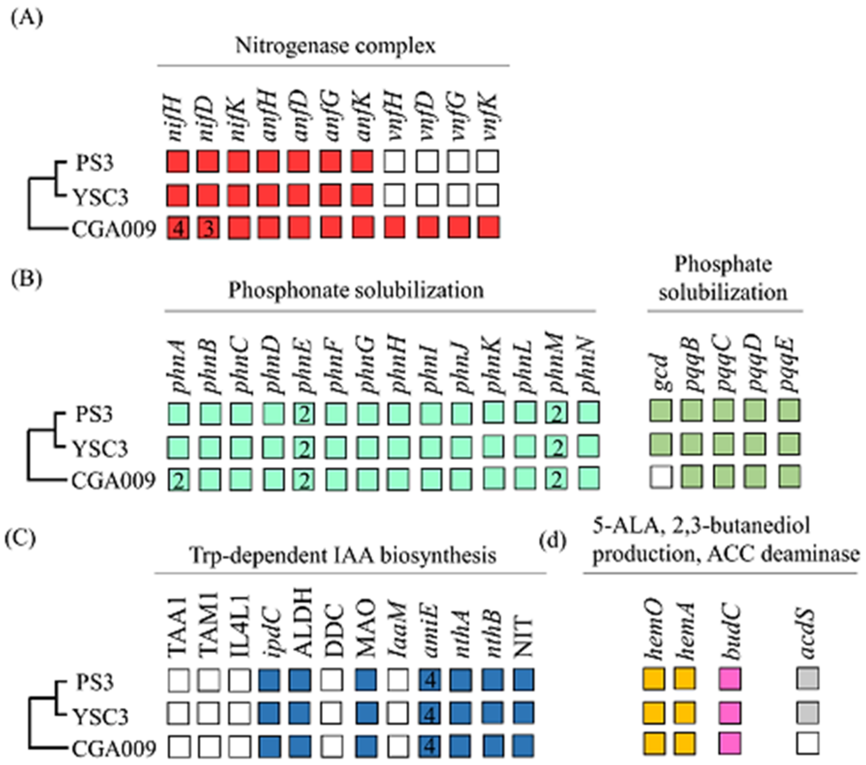

Supplementary Figure S1. Genes potentially related to plant growth promotion and biofilm formation in different strains of PS3, YSC3, and CGA009. An overview of 150 genes showing their presence (colored) and absence (empty) of (**A**) nitrogenases, (**B**) (**C**) phosphate solubilization, Trp-dependent IAA biosynthesis (**D**) 5-ALA, 2,3-butanediol production, ACC deaminase. Multicopies of genes are labeled by their copy number inside the filled squares. TAA1, tryptophan-pyruvate 155 aminotransferase; TAM1, tryptophan aminotransferase; IL4L1, amino-acid oxidase; 156 ALDH, aldehyde dehydrogenase; DDC, aromatic amino acid decarboxylase; TSO, 157 tryptophan side-chain oxidase; MAO, monoamine oxidase; NIT, nitrilase [40].

Supplementary Table S1. Beneficial effects of PNSB on different crops. Most of the information listed below was adapted from Table 1 and Table 2 of Sakarika et al., (2020) [39].

No.	Reference	Country	PNSB	Application Method	Dosage	Crop	Growth Conditions	Beneficial effect
1	Kobayashi and Haque (1971)	Japan	*R. capsulatus*	Irrigation	PNSB powder	Rice variety Kyoto Asahi	Pot	Enhanced grain number per ear by 32%
2	Kobayashi and Tchan (1973)	Japan, Australia	Phototrophic bacteria	Irrigation	Diluted solution	Citrus (10 years old)	Field	Increased fruit weight and sugar and carotenoid contents
3	Elbardy and Elbanna (1999)	Egypt	*R.capsulatus* DSM155	Irrigation	2.2 × 10^8 CFU/mL of suspension into 60 mL nutrient solution	Rice cv. Giza 159, 171, 176, 181	Hydroponic	Improved aerial part by 47–100%<br>Enhanced N content by 45–78%
4	Elbardy et al., 1999b	Egypt	*R. capsulatus* DSM155	Dipping	10^8 CFU/mL suspension mixed with 10% (*w/v*) gum arabic<br>0.05 M phosphate buffer (pH 7.0) mixed with 10% (*w/v*) gum arabic	Rice variety Giza 177	Pot	Increased straw yield and grain yield by 9–33%<br>Increased panicle length, panicle weight, and panicle number
5	Harada et al., 2005	Japan	*R. palustris* KN122	Irrigation	10 g rice straw with 1.7 × 10^{11} MPN/pot (day 0)<br>5.0 × 10^9 MPN/pot (day 43)<br>5.1 × 10^{10} cells/pot (day 86)	Rice variety Nipponbare	Pot	Increasing grain yield per pot by 21–29%<br>Increased plant height and tiller number
6	Koh and Song (2007)	China	*R. palustris* KL9 and BL6	Irrigation	4 × 10^9 cell suspension (daily for 8 weeks)	Tomato	Petri dish	Increased dry mass of seedlings<br>Increased the root and shoot lengths of germinated seedlings by 123.5% and 54%, respectively
7	Lee et al., 2008	Korea	*R. capsulatus* KL9 and BL6	Dipping	One application of 5 × 10^7 cells	Tomato	Pot	Increased fresh weight by 97% compared with that of the control<br>Increased lycopene content and vegetative growth (root and shoot)
8	Kondo et al., 2010	Japan	*R. sphaeroides*	Irrigation (split)	2.5–1.25 g cells	Tomato	Field	No significant difference in fruit weight but increased contents of ascorbic acid, malic acid and phosphoric acid in fruit
9	Eldin and Elbanna (2011)	Egypt	*R. sphaeroides* DSM155	Irrigation	10^8 CFU/mL cell suspension with 10% of gum arabic	Rice cv. Giza 177	Field	Improved grain yield up to 66%<br>Increased reproductive parameters in paddy

10	Yin et al., 2012	China	*PSB*	Foliar spraying	2.0 × 10^9 CFU/mL twice per week	Chinese dwarf cherry	Hydroponic (drought stress)	Significant accumulation of fresh weight, leaf area and photosynthetic rate
								Induced the activities of superoxide dismutase (SOD) and ascorbate peroxidase (APX)
11	Wu et al., 2013	China	*Rhodopseudomonas* sp. ISP-1	Foliar spraying	Foliar spraying (F): 2.4 × 10^{12} CFU (total)	Sweet leaves	Pot	Increased the plant yield in all treatments and increased the chlorophyll content, shoot biomass and stevioside content significantly in the foliar with spray treatment.
				Irrigation	Soil irrigation (S): 3.0 × 10^{12} CFU (total) Foliar spraying and soil irrigation (F+S): 2.7 × 10^{12} CFU (total)			
12	Kang et al., 2014	Korea	*R. sphaeroides* KE149	Irrigation	5-mL cell suspensions (1 × 106 CFU/mL)	Cucumber	Pot (water lodging stress)	Increased root length, shoot fresh weight, shoot dry weight, and chlorophyll content via secretion of indole acetic and organic acids
								Enhanced on giberellin content in plant Reduction on abscisic acid content in cucumber
13	Wong et al., 2014	Taiwan	*R. palustris* PS3 and YSC3	Irrigation	10^6 CFU/g per week	Maruba santoh	Pot	PS3 and half the normal amount of fertilizer produced the same plant growth potential as 100% of the normal amount of fertilizer
14	Yang et al., 2014	China	*Ru. gelatinosus*	Irrigation	Adjust bacterial concentration to an OD_{600} of 0.5 and 10 mL inoculated into soil	Tomato	Field	Increased agricultural traits and antioxidant value of tomato
15	Miao et al., 2014	China	Photosynthetic bacteria (PSB)	Foliar spraying	1×10^9–4×10^9 CFU/mL	Yellow-skinned watermelon	Field (low temperature stress)	Decreased the decomposition of chlorophyll and antioxidant enzymes to protect the membrane system under low temperature stress
16	Ge and Zhang, 2015	China	PS21(*Rhodopseudomonas* sp., *Rhodospirillum* sp.)	Irrigation	1 × 10^7 CFU/mL	Wheat	Pot (tetrabromobisphenol A stress)	Increased soluble sugar content, soluble protein content, SOD, CAT and POD activities
								Decreased MDA content in wheat
17	Hsu et al., 2016	Taiwan	*R. palustris* PS3 and YSC3	Irrigation	10^6 CFU/mL per week	Maruba santoh	Hydroponic	Increased the number of consumable leaves by 28% and reduced the nitrate content in Chinese cabbage
						Cuiyu cabbage		Increased the number of consumable leaves by 40% and reduced the nitrate content in Cuiyu cabbage

						Crinkle garden lettuce		Increased plant weight by 16% and reduced the nitrate content in lettuce
18	Kantha et al., 2015	Thailand	*R. palustris* TN114, PP803, and TK103	Irrigation	One application every two weeks of 10^8 cells/gram with rice husk ash and rice straw (vegetative growth)	Rice	Saline field	Increased grain yield by 5–8% (inoculated with PP803 and TK103)
						(Thai jasmine KDML105)	Organic field	Increased grain yield by 30–48% when inoculated with TN114, TK103 and PP803 compared with the control
					One application per week of 10^8 cells/gram with rice husk ash and rice straw (reproductive and maturation stages)			
19	Kantachote et al., 2016	Thailand	*R. palustris* TN114 and PP803	Root dipping	Immerse seedlings in culture supernatant for 12 hours	Rice	Pot	Increased fresh and dry weights by up to 70% in both saline and normal conditions (inoculation treatment)
20	Xu et al., 2016	China	*R. palustris* sp.	Irrigation	Irrigate *Stevia* seedlings with 10^7 CFU/mL x 100 mL inoculant on 60th, 67th, 74th, and 81st day after emergence	Sweet leaf	Field	Increased biomass slightly but not significantly
21	Lee et al., 2016	Taiwan	*R. palustris* PS3	Irrigation	Storage formulated inoculant applied into 300 g of soil per week	Maruba santoh	Pot	Promoted fresh and dry weight of shoot by 40–56%
22	Batool et al., 2017	Pakistan	*R. palustris* CS2 and SS5	Seed bacterization	Adjust bacterial concentration to an OD_{600} of 0.5 and dip seeds in bacterial inoculum for 15-20 minutes	*Vigna mungo*	Pot (Arsenic stress)	Beneficial bacteria help to remediate soil containing arsenic; plant shoot and root lengths increased up to 12–24%.
23	Su et al., 2017	China	*R. palustris* GJ-22	Foliar spraying	Apply 6 × 10^7 CFU/mL solution to the leaves	Tobacco	Pot	Improved on IAA production and ISR response<br>Increased the number of first-class tobacco leaves by 27–40%<br>Enhanced seed germination rate
24	Nookongbut et al., 2018	Thailand	*R. palustris* C1	Irrigation	1 × 10^8 cells/mL (inoculation every week for 30 days)	Rice (HomNil and PathumThani)	Hydroponic (Arsenic stress)	Reduced rice seedling loss by 50% through amelioration of arsenic toxicity in water
		Australia	*Ru.benzoatilyticus* C31					Improved vegetative growth and increased germination index by up to 10 times compared with that in other treatments
25	Imsong et al., 2018	India	Photosynthetic bacteria	Irrigation	50–150 L/ hectare of cell suspension	Broccoli	Field	Increased yield, quality and growth parameters

26	Khuong et al., 2018	Vietnam	*R. palustris* TLS06, VNW02, NNW64, and VNS89	Irrigation	5.4 x 10^4 cells/g DSW in 6 kg soil/pot	Rice	Pot (low available phosphorus soil)	Increased rice grain yield by 26.5%<br>Improved plant height, panicle length, and the spikelet/panicle ratio
27	Luo et al., 2019	China	*R. palustris* PSB06 and CGA009	Irrigation	Soak seeds for 3 h + root irrigation (15 days after transplanting)	Rice (LiangYou number 7)	Pot	PSB treatment (Irrigation):<br>Increased rice stalks by 6–17%; Increased peroxidase (POD), and superoxide dismutase (SOD) activities
				Seed inoculation	Soak seeds for 3 h			CGA009 treatment (seed inoculation): Increased rice stalks by approximately 5–30%;<br>Increased SOD activity
28	Ge and Zhang (2019)	China	*R. palustris* G5	Irrigation	25 mL per pot (10^8 CFU/mL)	Cucumber	Pot (salt stress)	Increased shoot height, root length, and plant fresh and dry weight
29	Nookongbut et al., 2020	Thailand, Vietnam	*R. palustris* KTSSR54	Irrigation	Suspend 10^8 cfu/mL (OD = 0.5) of inoculum in 3 L of tap water every two weeks	Rice	Pot	Reduced grain loss (100-grain weight) by 15–40% under field conditions with biotic stress
30	Zhang et al., 2020	China	*R. palustris* GJ-22	Foliar spraying	6.79 x 10^6 or 19 x 10^7 CFU/mL on 3rd to 4th leaf, total volume is 50 mL per pot	Potato tuber Xingjia2	Pot	Reduced late blight disease through biocontrol
31	Kang et al., 2020	Korea	*R. sphaeroides* KE149	Irrigation	50 mL of cell suspension (1 × 10^8 CFU/mL) for 7 days	Adzuki bean	Pot (water stress)	Decreased stress-responsive endogenous abscisic acid and jasmonic acid and increased salicylic acid<br>Increased the levels of calcium (Ca), magnesium (Mg), and potassium (K)<br>Increased root length, shoot length, stem diameter, biomass, and higher chlorophyll content
32	Lo et al., 2020	Taiwan	*R. palustris* sp. PS3 and YSC3	Irrigation	250 mL of modified cell suspension medium (10^8 CFU/mL) into 30 L of tank containing nutrient solution per week	Cuiyu cabbage	Hydroponic	Promoted shoot and root weight by 12–48%
					2 mL of modified cell suspension medium (10^8 CFU/mL) into 300 g of soil per week		Pot	

33	Xiao et al., 2021	China	*R. palustris* sp.	Irrigation	Apply 24 g/m^2 of inoculum in the tillering stage	Rice	Pot	Increased rice yields by 9.84–17.73%
34	Kang et al., 2021		*R. sphaeroides* KE149	Irrigation	10 mL of cell suspension (10^8 CFU/mL)	Soybean	Pot (water logging stress)	Improved plant morphological attributes, such as root length, shoot length, and fresh biomass.<br>Increased jasmonic acid, phenolic and flavonoids contents<br>Enhanced the levels of endogenous phytohormones (such as abscisic acid) under water lodging conditions<br>Decreased the levels of endogenous phytohormones (such as abscisic acid) under normal conditions
35	Hsu et al., 2021	Taiwan	*R. palustris* sp. PS3 and YSC3	Irrigation	10^6 CFU/mL per week	Maruba santoh	Hydroponic	Increased cabbage fresh weight by 50% compared with that of the control<br>Increased consumable leaf expansion and cell plate number
36	Sakpirom et al., 2021	Thailand	R. palustris TN110 and Ru.gelatinosus TN414	Irrigation	1 × 10^8 CFU/mL; 1 × 10^7 CFU/mL	Paddy	Pot	Both liquid and solid formulations increased rice growth and parameters in paddy soil compared with controls.
37	Liu et al., 2021	China	*R. sphaeroides*	Irrigation	1 × 10^7 CFU/g per pot<br>-	Oilseed rape	Pot	Increased the plant total N<br>Recruit beneficial diazotrophic rhizobacteria
38	Wang et al., 2021	China	*R. palustris* ISP-1	Irrigation	Adjust to 10^8 CFU/mL and 300 L/ha of suspensions for twice, sowing and pegging stage	Peanut	Field	Increased 12.5% of total peanut yield and nutrient content in soil

Highlighted in dark grey represent the updated references not included in Sarkarika et al., 2020: #10, #12, #14, #15, #16, #21, #25, #27, #28, #29, #31, #32, #33, #34, #35, #36, #37, #38.

Supplementary Table S2. Plant growth responses to PS3 inoculation under different experimental conditions and farming systems.

Farming System	Plant Species	Application Method	Dosage (CFU/g soil)	Instructions	Inoculations	Effects
Conventional	Heading Chinese cabbage	Irrigation	10^5–10^6	20 mL per plant	8	yield increased by ~39%
Organic	Chinese cabbage	Irrigation	10^5–10^7	200 L per hectare	1	yield increased by ~43%
	Lettuce	Irrigation	10^5–10^7	200 L per hectare	1	yield increased by ~30%
	Sesame leaves	Irrigation	10^5–10^7	200 L per hectare	1	yield increased by 25–30%
	Tomato	Irrigation	10^5–10^7	200 mL per plant	3	yield increased by ~25% and ~38% in fruit fresh and dry weights, respectively; sugar/acid ratio increased by 45%; vitamin C and lycopene contents increased by 25%
Conventional with organic amendment	Chinese flowering cabbage (choi sum)	Irrigation	10^5–10^7	200 L per hectare	1	yield increased by ~27%
	Pepper leaves	Irrigation	10^5–10^7	200 L per hectare	1	yield increased by ~26%

Reference

1. Kobayashi, M.; Haque, M. Z., Contribution to nitrogen fixation and soil fertility by photosynthetic bacteria. *Plant Soil* **1971,** *35* (1), 443-456.
2. Kobayashi, M.; Tchan, Y. T., Treatment of industrial waste solutions and production of useful by-products using a photosynthetic bacterial method. *Water Research* **1973,** *7* (8), 1219-1224.
3. Elbadry, M.; El-Bassel, A.; Elbanna, K., Occurrence and dynamics of phototrophic purple nonsulphur bacteria compared with other asymbiotic nitrogen fixers in ricefields of Egypt. *World Journal of Microbiology and Biotechnology* **1999,** *15* (3), 359-362.
4. Elbadry, M.; Gamal-Eldin, H.; Elbanna, K., Effects of *Rhodobacter capsulatus* inoculation in combination with graded levels of nitrogen fertilizer on growth and yield of rice in pots and lysimeter experiments. *World J. Microbiol. Biotechnol.* **1999,** *15*, 393-395.
5. Harada, N.; Nishiyama, M.; Otsuka, S.; Matsumoto, S., Effects of inoculation of phototrophic purple bacteria on grain yield of rice and nitrogenase activity of paddy soil in a pot experiment. *Soil Science & Plant Nutrition* **2005,** *51* (3), 361-367.
6. Koh, R.-H.; Song, H.-G., Effects of application of *Rhodopseudomonas* sp. on seed germination and growth of tomato under axenic conditions. *J. Microbiol. Biotechnol.* **2007,** *17*, 1805-10.
7. Lee, K.-H.; Koh, R.-H.; Song, H.-G., Enhancement of growth and yield of tomato by *Rhodopseudomonas* sp. under greenhouse conditions. *The Journal of Microbiology* **2008,** *46* (6), 641-646.
8. Kondo, K.; Nakata, N.; Nishihara, E., Effect of the purple non-sulfur bacterium (*Rhodobacter sphaeroides*) on the Brix, titratable acidity, ascorbic acid, organic acid, lycopene and β-carotene in tomato fruit. *Journal of Food, Agriculture & Environment* **2010,** *8* (2 part 2), 743-746.
9. Gamal-Eldin, H.; Elbanna, K., Field evidence for the potential of *Rhodobacter capsulatus* as biofertilizer for flooded rice. *Curr Microbiol* **2011,** *62* (2), 391-5.
10. Yin, Z.; Shang, Z.; Wei, C.; Ren, J.; Song, X., Foliar sprays of photosynthetic bacteria improve the growth and anti-oxidative capability on Chinese dwarf cherry seedlings. *Journal of Plant Nutrition - J PLANT NUTR* **2012,** *35*, 840-853.
11. Wu, Y.; Jin, X.; Liao, W.; Hu, L.; Dawuda, M. M.; Zhao, X.; Tang, Z.; Gong, T.; Yu, J., 5-Aminolevulinic Acid (ALA) alleviated salinity stress in cucumber seedlings by enhancing chlorophyll synthesis pathway. *Frontiers in Plant Science* **2018,** *9* (635).
12. Kang, S.-M.; Radhakrishnan, R.; You, Y.-H.; Khan, A.; Park, J.-M.; Lee, S.-M.; Lee, I.-J.; Francis, T., Cucumber performance is improved by inoculation with plant growth-promoting microorganisms. *Acta Agriculturae Scandinavica, Section B - Soil & Plant Science* **2014,** *65*.
13. Wong, W. T.; Tseng, C. H.; Hsu, S. H.; Lur, H. S.; Mo, C. W.; Huang, C. N.; Hsu, S. C.; Lee, K. T.; Liu, C. T., Promoting effects of a single *Rhodopseudomonas palustris* inoculant on plant growth by *Brassica rapa chinensis* under low fertilizer input. *Microbes Environ* **2014,** *29* (3), 303-13.

14. Yang, F.; Tian, J. Y.; Yang, P. P.; Feng, H.; He, G. S.; Chen, X. D.; Lu, Y. S.; Peng, G. X., Effects of inoculant of photosynthetic bacteria on tomato quality, soil fertility and soil microbial characteristics. *Journal of South China Agricultural University* **2014,** *35* (1), 49-54.
15. Miao, J.s. L., J.; Liu, Y.l.; Wu, J.f., Effect of photosynthetic bacterial on photosynthesis and antioxidant enzyme system of watermelon seedlings in early spring. *Northern Horticulture 2014* (21), 42-44.
16. Ge, H.-L.; Zhang, F.-L., The effects of composite photosynthetic bacterial inoculant PS21 on the biochemical characteristics of wheat seedlings under tetrabromobisphenol A stress. *Biotechnology & Biotechnological Equipment* **2015,** *29*, 1-10.
17. Hsu, S. L., K.; Fang, W.; Lur, H.; Liu, C.,, Application of phototrophic bacterial inoculant to reduce nitrate content in hydroponic leafy vegetables. *Crop Environ. Bioinf.* **2015,** *12* (1), 30-41.
18. Kantha, T.; Kantachote, D.; Klongdee, N., Potential of biofertilizers from selected *Rhodopseudomonas palustris* strains to assist rice (*Oryza sativa* L. subsp. indica) growth under salt stress and to reduce greenhouse gas emissions. *Annals of microbiology* **2015,** *65* (4), 2109-2118.
19. Kantachote, D.; Nunkaew, T.; Kantha, T.; Chaiprapat, S., Biofertilizers from *Rhodopseudomonas palustris* strains to enhance rice yields and reduce methane emissions. *Applied Soil Ecology* **2016,** *100*, 154-161.
20. Xu, J.; Feng, Y.; Wang, Y.; Luo, X.; Tang, J.; Lin, X., The foliar spray of *Rhodopseudomonas palustris* grown under *Stevia* residue extract promotes plant growth via changing soil microbial community. *Journal of Soils and Sediments* **2016,** *16* (3), 916-923.
21. Lee, S.-K.; Lur, H.-s.; Lo, K.-J.; Cheng, K.-C.; Chuang, C. C.; Tang, S.-J.; Yang, Z.-w.; Liu, C.-T., Evaluation of the effects of different liquid inoculant formulations on the survival and plant-growth-promoting efficiency of *Rhodopseudomonas palustris* strain PS3. *Appl. Microbiol. Biotechnol.* **2016,** *100*, 7977-7987.
22. Batool, K.; tuz Zahra, F.; Rehman, Y., Arsenic-redox transformation and plant growth promotion by purple nonsulfur bacteria *Rhodopseudomonas palustris* CS2 and *Rhodopseudomonas faecalis* SS5. *BioMed Res. Int.* **2017,** *2017*, 6250327.
23. Su, P.; Tan, X.; Li, C.; Zhang, D.; Cheng, J.; Zhang, S.; Zhou, X.; Yan, Q.; Peng, J.; Zhang, Z.; Liu, Y.; Lu, X., Photosynthetic bacterium *Rhodopseudomonas palustris* GJ-22 induces systemic resistance against viruses. *Microb Biotechnol* **2017,** *10* (3), 612-624.
24. Nookongbut, P.; Kantachote, D.; Nguyen Quoc, K.; Khuong, Q.; Sukhoom, A.; Tantirungkij, M.; Limtong, S., Selection of acid-resistant purple nonsulfur bacteria from peat swamp forests to apply as biofertilizers and biocontrol agents. *Journal of Soil Science and Plant Nutrition* **2019,** *19*.
25. Imsong, T.; Bahadur, V.; Topno, S. E., Influence of photosynthetic bacteria and Biochar on the growth,yield and quality of broccoli (*Brassica olearacea* var. italica). *Journal of Pharmacognosy and Phytochemistry* **2018,** *7* (3), 3722-372.
26. Khuong, N. Q.; Kantachote, D.; Onthong, J.; Xuan, L. N. T.; Sukhoom, A., Enhancement of rice growth and yield in actual acid sulfate soils by potent acid-resistant *Rhodopseudomonas palustris* strains for producing safe rice. *Plant Soil* **2018,** *429* (1), 483-501.
27. Luo, L.; Wang, P.; Zhai, Z.; Su, P.; Tan, X.; Zhang, D.; Zhang, Z.; Liu, Y., The effects of *Rhodopseudomonas palustris* PSB06 and CGA009 with different agricultural applications on rice growth and rhizosphere bacterial communities. *AMB Express* **2019,** *9* (1), 173.
28. Ge, H.; Zhang, F., Growth-promoting ability of *Rhodopseudomonas palustris* G5 and its effect on induced resistance in cucumber against salt stress. *Journal of Plant Growth Regulation* **2019,** *38* (1), 180-188.
29. Nookongbut, P.; Kantachote, D.; Nguyen Quoc, K.; Tantirungkij, M., The biocontrol potential of acid-resistant *Rhodopseudomonas palustris* KTSSR54 and its exopolymeric substances against rice fungal pathogens to enhance rice growth and yield. *Biol. Control* **2020,** *150*, 104354.
30. Zhang, X.; Li, X.; Zhang, Y.; Chen, Y.; Tan, X.; Su, P.; Zhang, D.; Liu, Y., Integrated control of potato late blight with a combination of the photosynthetic bacterium *Rhodopseudomonas palustris* strain GJ-22 and fungicides. *Biocontrol* **2020,** *65*, 635-645.
31. Kang, S. M.; Adhikari, A.; Lee, K. E.; Khan, M. A.; Khan, A. L.; Shahzad, R.; Dhungana, S. K.; Lee, I. J., Inoculation with indole-3-acetic acid-producing rhizospheric *Rhodobacter sphaeroides* KE149 augments growth of Adzuki bean plants under water stress. *Journal of microbiology and biotechnology* **2020,** *30* (5), 717-725.
32. Lo, K.-J.; Lee, S.-K.; Liu, C.-T., Development of a low-cost culture medium for the rapid production of plant growth-promoting *Rhodopseudomonas palustris* strain PS3. *PLoS One* **2020,** *15* (7), e0236739.
33. Xiao, X.; Zhu, Y.; Gao, Y.; Fu, J.; Zhao, Y.; Zhao, L., Inoculation of paddy soils with *Rhodopseudomonas palustris* enhanced heavy metal immobilisation. *Plant, Soil and Environment* **2021,** *67*.
34. Kang, S.-M.; Adhikari, A.; Khan, M. A.; Kwon, E.-H.; Park, Y.-S.; Lee, I.-J., Influence of the rhizobacterium *Rhodobacter sphaeroides* KE149 and biochar on waterlogging stress tolerance in *Glycine max* L. *Environments* **2021,** *8* (9), 94.
35. Hsu, S.-H.; Shen, M.-W.; Chen, J.-C.; Lur, H.-S.; Liu, C.-T., The photosynthetic bacterium *Rhodopseudomonas palustris* strain PS3 exerts plant growth-promoting effects by stimulating nitrogen uptake and elevating auxin levels in expanding leaves. *Front. Plant. Sci.* **2021,** *12* (93).
36. Sakpirom, J.; Nunkaew, T.; Khan, E.; Kantachote, D., Optimization of carriers and packaging for effective biofertilizers to enhance *Oryza sativa* L. growth in paddy soil. *Rhizosphere* **2021,** *19*, 100383.
37. Liu, Y.; Gao, J.; Bai, Z.; Wu, S.; Li, X.; Wang, N.; Du, X.; Fan, H.; Zhuang, G.; Bohu, T.; Zhuang, X., Unraveling mechanisms and impact of microbial recruitment on oilseed rape (*Brassica napus* L.) and the rhizosphere mediated by plant growth-promoting rhizobacteria. *Microorganisms* **2021,** *9* (1).

38. Wang, Y.; Peng, S.; Hua, Q.; Qiu, C.; Wu, P.; Liu, X.; Lin, X., The long-term effects of using phosphate-solubilizing bacteria and photosynthetic bacteria as biofertilizers on peanut yield and soil bacteria community. *Front Microbiol* **2021,** *12*, 693535.
39. Sakarika, M.; Spanoghe, J.; Sui, Y.; Wambacq, E.; Grunert, O.; Haesaert, G.; Spiller, M.; Vlaeminck, S.E. Purple non-sulphur bacteria and plant production: Benefits for fertilization, stress resistance and the environment. *Microb. Biotechnol.* **2020**, *13*, 1336–1365. https://doi.org/10.1111/1751-7915.13474.
40. Lo, K.-J.; Lin, S.-S.; Lu, C.-W.; Kuo, C.-H.; Liu, C.-T. Whole-genome sequencing and comparative analysis of two plant-associated strains of Rhodopseudomonas palustris (PS3 and YSC3). *Sci. Rep.* **2018**, *8*, 1–15.
